# Galapagos III World Evolution Summit: why evolution matters

**DOI:** 10.1186/1936-6434-6-28

**Published:** 2013-09-24

**Authors:** Guillermo Paz-y-Miño-C, Avelina Espinosa

**Affiliations:** 1 Department of Biology, University of Massachusetts Dartmouth, 285 Old Westport Road, North Dartmouth, Massachusetts 02747-2300, USA; 2 Department of Biology, Roger Williams University, One Old Ferry Road, Bristol, Rhode Island 02809, USA

**Keywords:** ENCODE, Holobionts, Incompatibility hypothesis, Introgressive descent, Microbiosphere, Multilevel selection, Origin of life, Protoribosome, Species crypticity, Virosphere

## Abstract

There is no place on Earth like the Galapagos Islands and no better destination to discuss the reality of evolution. Under the theme ‘Why Does Evolution Matter’, the University San Francisco of Quito (USFQ), Ecuador, and its Galapagos Institute for the Arts and Sciences (GAIAS), organized the III World Evolution Summit in San Cristóbal Island. The 200-attendee meeting took place on 1 to 5 June 2013; it included 12 keynote speakers, 20 oral presentations by international scholars, and 31 posters by faculty, postdocs, and graduate and undergraduate students. The Summit encompassed five sessions: evolution and society, pre-cellular evolution and the RNA world, behavior and environment, genome, and microbes and diseases. USFQ and GAIAS launched officially the Lynn Margulis Center for Evolutionary Biology and showcased the Galapagos Science Center, in San Cristóbal, an impressive research facility conceptualized in partnership with the University of North Carolina at Chapel Hill, USA. USFQ and GAIAS excelled at managing the conference with exceptional vision and at highlighting the relevance of Galapagos in the history of modern evolutionary thinking; Charles Darwin’s visit to this volcanic archipelago in 1835 unfolded unprecedented scientific interest in what today is a matchless World Heritage.

## Background

The Galapagos World Evolution Summit reverberates every four years and this past 1 to 5 June 2013, University San Francisco of Quito (USFQ), Ecuador, and its Galapagos Institute for the Arts and Sciences (GAIAS) organized it for the third time. The Galapagos volcanic archipelago might be distantly located 900 km west of the coasts of Ecuador, but the Summit was constantly ‘close by’ in the news and social media. Instant tweeting, video uploading online, TV and radio reporters chasing the speakers, and frequent press releases made this scientific event uniquely dynamic.

Under the scope ‘Why Does Evolution Matter’, the 200-attendee Summit took place at the Charles Darwin Center in Puerto Baquerizo Moreno, San Cristóbal Island. It included 12 keynote speakers, 20 oral presentations by international scholars, and 31 posters by faculty, postdocs, and graduate and undergraduate students. The discussions encompassed five sessions: evolution and society, pre-cellular evolution and the RNA world, behavior and environment, genome, and microbes and diseases.

Carlos Montúfar (Provost USFQ) opened the Summit by connecting Ecuador and Galapagos with two major transformations in scientific thinking, both intertwined with the history of what today are Ecuador and its Galapagos National Park (Montúfar 2013). First, a paradigm shift from a Cartesian to a Newtonian view of the planet. And, second, the replacement of creationism and the Victorian concept of ‘species’ immutability’ with Darwin’s Theory of Evolution by means of natural selection (Darwin 1859, 1871). In retrospect, Montúfar explained, Darwin’s interest in traveling the world on board of the HMS Beagle (1831–1836), and subsequent visit to the Galapagos Islands in 1835, was inspired by Alexander Von Humboldt’s *Personal Narrative of Travels to the Equinoctial Regions of America* (1799–1804), a recount of expeditions and scientific discoveries in pristine Caribbean and South American landscapes. ‘I formerly admired Humboldt, I now almost adore him; he alone gives any notion, of the feelings which are raised in the mind on first entering the Tropics …’ Darwin wrote in 1832 in a letter to botanist John Stevens Henslow (Darwin Correspondence Project 2013). Fascinatingly, Humboldt’s adventurous traveling had been inspired by those of the French geographer Charles Marie de la Condamine who in the 1730 s led the geodesic mission to measure the length of a meridian arc at the equator, in present Ecuadorian Andes, and thus test Issac Newton’s postulate that the Earth, due to gravitational forces and its rotation at a stable angle in respect to an orbiting trajectory around the sun, should be an oblate spheroid flattened at the poles. By comparatively measuring the arc at the equator (la Condamine’s team) and at the North Pole (another team sponsored as well by the Paris Academy of Sciences), Newton’s laws of motion and universal gravitation conceived in his *Mathematical Principles of Natural Philosophy* (Newton 1687) were reconfirmed and the two-centuries ruling model of our planet’s elongation at the poles was debunked. But the intertwined history of scientific events continues: the Newtonian proposal was rooted in Nicolaus Copernicus heliocentric hypothesis (= the sun as center of our solar system), confirmed by Galileo Galieli and Johannes Kepler (iconic contributors to astronomy in the 1600 s), prior debunkers of the geocentric, creationist view (= the Earth as center of the universe). A major scientific paradigm shift, indeed! And thus Montúfar linked, with hindsight, Darwin’s visit to the Galapagos archipelago with the foundations of modern science, from the Copernican revolution (1500 s) to the Darwinian revolution (1800 s).

## Main text

### The Summit’s sessions and keynote addresses

#### Evolution and society

The core discussion about ‘why evolution matters’ was exemplified by this provocative session on the patterns of low acceptance of evolution worldwide. Guillermo Paz-y-Miño-C (University of Massachusetts Dartmouth, USA) and Avelina Espinosa (Roger Williams University, USA) coauthored the keynote address ‘Evolution, Science, Pseudo Science and the Public’s Perception of Reality, in ‘ which they postulated that the controversy evolution versus creationism (including all its modern forms: theistic evolution, creation science, young-earth creationism, Intelligent Design, BioLogos) is intrinsic to the incompatibility between scientific rationalism/empiricism and the belief in supernatural causation (Paz-y-Miño-C and Espinosa 2013a, b). To test the ‘incompatibility hypothesis,’ these authors have conceptualized a Cartesian landscape where the dependent variable acceptance of evolution is plotted as function of three factors: level of understanding the essence of science, familiarity with the concept of evolution, and personal belief convictions. By scientifically polling human subjects of diverse educational attainment, from highly educated professors to in-the-process-of-acquiring-education college students, Paz-y-Miño-C and Espinosa (2013a, b) have demonstrated significant associations: open acceptance of evolution increased with subject academic level, from college students (63%) to educators of prospective teachers (72%) and to researchers in academia (94%); in all groups (grand total *n* = 1,392), understanding science and evolution were positively correlated (that is those scoring high in understanding science S, on a scale of 0 to 3 points, from least to most, also scored high in familiarity with evolution E, as follows: students S = 1.80 *vs.* E = 1.60, educators of prospective teachers S = 1.98 *vs.* E = 1.77, researchers in academia S = 2.49 *vs.* E = 2.49), but level of understanding science or evolution decreased with increasing religiosity R (negative association of variables) and the least educated were the most religious (students R = 0.89, educators of prospective teachers R = 1.31, researchers in academia R = 0.49; Paz-y-Miño-C and Espinosa 2013a, b). Interestingly, Paz-y-Miño-C and Espinosa documented that non-religious participants in their studies (R = 0.0), who did not possess the academic credentials of the research scholars, have shown levels of understanding the foundations of science and evolution comparable to those of the highly educated professors (that is, atheists, non-believers, and agnostics S = 2.34, E = 2.41, R = 0.0, sample *n* = 133) (Paz-y-Miño-C and Espinosa 2013b).

Paz-y-Miño-C and Espinosa also expressed concern about the international patterns of acceptance of evolution: only 41% of adults worldwide (24 countries, *n* = 18,829) accept evolution, and they do it under the premise that a deity created humans; 31% do not know who to trust in matters of evolution, neither scientists nor spiritualists; and 28% are strict creationists who believe in religious scriptures concerning the origin of our universe and of humans (for example, Genesis: the creation of the universe by God a few thousand years ago = Young Earth Creationists), and explicitly reject the fact that humans are apes (IPSOS 2011; Paz-y-Miño-C and Espinosa 2012, 2013b). Geographically, world Christians in six continents (North America 30%; South America 30%; Europe and Great Britain 50%; Asia 30%; Africa 25%; and Australia 30%; data extracted from Wilson 2010) accept evolution more than Muslims in Turkey (19–22%), Indonesia (11–16%), Pakistan (14%), Malaysia (12%), and Egypt (8%), except for Kazakhstan (38%) (data extracted from IPSOS 2011 and Hameed 2008). What is crucial to deduce from these international trends, emphasized Paz-y-Miño-C and Espinosa, is that religiosity, all over the world, interferes with acceptance of evolution, and that the negative correlation between religiosity and science/evolution literacy is inherent to the incompatible nature between supernatural causation and empirical reality (Paz-y-Miño-C and Espinosa 2012, 2013b).

### Pre-cellular evolution and the RNA world

Nobel laureate in Chemistry, 2009, Ada Yonath (Weizmann Institute, Israel), Marie-Christine Maurel (University Pierre-and-Marie-Curie, France), and Antonio Lazcano (National Autonomous University of Mexico) offered a brilliant session about the ribonucleic acid (RNA) and the environment in which this molecule probably originated. Indeed, the emergence of an RNA-like entity capable of synthesizing peptides was a significant development prior to the last universal common ancestor of life; Yonath’s team has hypothesized the existence of such entity and called it ‘protoribosome’ (Fox et al. 2012). The protoribosome probably possessed a site for peptide bond formation, or ‘cavity’ (still a highly conserved region in ribosomes of modern archaea, bacteria, and eukarya), and an adjacent passage that evolved into the modern ribosomal exit tunnel, a crucial structure for peptide polymerization. Yonath suggested that when activated amino acids (for example, bound to small RNAs, analogous to modern tRNAs) were in proximity to the ‘cavity, attracted by natural polarity, a peptide ‘ bond could have formed spontaneously at the ‘active site’ of the protoribosome. The presence of the peptide in the ‘cavity’ would have prolonged its chemical stability and lifetime; subsequent addition of amino acids would have increased the overall stability of the oligopeptide formation, a feature favored by selection (Fox et al. 2012). This ‘protoribosome and tunnel’ hypothesis, therefore, offers an explanation for the emergence of protein synthesis and helps us envision a plausible scenario for the evolution of early stages in the genetic code; its implications for the origin of life are significant.

How would prebiotic chemistry of the ancient world evolve into early biology and the genetic-code/enzyme-mediated modern life? Marie Christine Maurel explained that the ‘RNA world hypothesis’ provides the conceptual framework to address this question. Half a century ago, Alexander Rich suggested that archaic self-replicating macromolecules with both encoding and catalyzing properties gave origin to the DNA-to-RNA-to-protein chemistry of today’s life (Neveu et al. 2013). To test an aspect of this proposal, Maurel’s team has experimented with ‘ribozymes’ (that is, highly conserved ribonucleic acid catalysts discovered in the 1980s which have such dual properties) under extreme conditions of pressure or temperature, which mimic the young Earth’s environments, and found high resilience in these RNA-catalysts to such conditions. Since ribozymes occur ubiquitously across taxa, they are relics of ancient biochemistry (Talini et al. 2009). Modern ribozymes mediate RNA processing reactions, including synthesis of messenger, transfer, and ribosomal RNAs; the functional component of the ribosome (as discussed by Ada Yonath, above) is, in principle, a ribozyme (a fascinating link between Maurel’s and Yonath’s keynote addresses). To further connect the modern with the ancient RNA world, Maurel has also exposed RNA-viroids (that is, vestiges of ancient life) to extreme environmental conditions and documented tolerance to variations in pressure and temperature (Kaddour et al. 2011; El-Murr et al. 2012); in addition, Maurel has demonstrated that viroids replicate in non-specific hosts, thus suggesting wide adaptability to dynamic environments and, therefore, potential survivability of RNAs over eons.

Antonio Lazcano also sympathized with the RNA world hypothesis and reviewed comprehensive evidence in its support, emphasizing on the catalytic properties of RNA (that is, ribozymes) and its evolutionary relevance for the origin of protein biosynthesis (for a historical recount leading to the formulation of the RNA world hypothesis see Lazcano 2012). Lazcano highlighted that because coenzymes/cofactors (for example, S-adenosyl methionine (SAM), tetrahydrofolate (THF), flavin mononucleotide (FMN), thiamine pyrophosphate (TPP), and adenosylcobalamin (AdoCbl), as well as adenosine tri phosphate (ATP), nicotinamide adenine dinucleotide (NAD), nicotinamide adenine dinucleotide phosphate (NADP), flavin adenine dinucleotide (FAD), and coenzyme A (CoA)) are essential for molecular pathways in which RNA participates, they probably evolved as part of an ancestral RNA-based metabolic apparatus now common to all cells (= metabolic fossils; Reyes-Prieto et al. 2012). Prebiotic chemistry, or the ‘broth of life’, was probably nourished by carbonaceous chondrites (solar system remnants of materials from which Earth formed, some continue to arrive in our planet in meteorites; Cleaves 2010), rich in simple amino acids which, if catalyzed, did tend to polymerize in complex three-dimensional structures (today’s proteins). Inferentially, Lazcano rooted Yonath’s protoribosome and Maurel’s ribozymes (above) with the origin of the most rudimentary metabolism (Alifano et al. 1996), and by envisioning the incubation of life by a young Earth, Darwin’s writings became relevant again: ‘there is grandeur in this view of life… from so simple a beginning endless forms most beautiful and most wonderful have been, and are being, evolved’ (Darwin 1859).

### Behavior and environment

This session included the keynote addresses by Charles Snowdon (University of Wisconsin Madison, USA) and Patricia Parker (University of Missouri St. Louis, USA), a significant presentation by Carlos Valle (USFQ-GAIAS), and a closing keynote address by Forest Rohwer (San Diego State University, USA).

Charles Snowdon opened the behavior talks by examining an old, yet incompletely answered question: why should an individual cooperate to help another? It is intriguing that although natural selection maximizes reproductive success of, mostly, individuals, animals still invest resources to benefit others. Neither kin selection nor strict reciprocity, or punishment to prevent free-riders, seem to adequately characterize human cooperation, which is distinctive among apes. Snowdon proposed that looking at cooperation through converging evolution may be illuminating and, for that, he compared humans (who cooperate to breed) to the cooperative-breeding New World Callitrichid primates (Snowdon and Cronin 2007; Cronin et al. 2010). Cooperative breeders have the innate predisposition to behave prosocially, which enhances performance in socio-cognitive and problem-solving settings (Cronin et al. 2010). Marmosets and tamarins cooperate remarkably: they coordinate behavior to solve problems, share and donate food, teach to and facilitate social learning among youngsters, have long-lasting memories for social partners, share labor and joint attention to collective tasks. Callitrichids provide rewards to socio-sexual partners (for example, grooming, non-conceptive sexual exchange) more willingly than punishment to minimize free riding, which builds mutual trust. Snowdon highlighted that rather than restricting our attention to mainly apes in search for answers about the origins of human altruism and cooperation, we should also look at the convergent evolution of cooperation in phylogenetically more distant species (that is, cooperative breeding hypothesis; Hrdy 2005); in essence, a plea for proper application of the comparative scientific method.

Patricia Parker discussed the impact of pathogens on the behavior, ecology, and conservation of the endemic avifauna in the Galapagos. Her phylogenetic (ancestry) and phylogeographic (distributional geographic patterns of ancestry) analyses suggest the existence of three categories of pathogens: those that arrived to the islands with their colonizing host(s) and diversified in parallel with it (them), those that switched to other hosts after arrival, and those associated with human colonization, which are of particular conservation concern (Parker and Whiteman 2012). Because bird extinctions have been documented in oceanic archipelagos (for example, Hawaii) after the arrival of avian malaria and pox virus (via introduction of domestic animals), Parker alerted that a comparable scenario is conceivable in the Galapagos since geographically long-isolated bird populations are particularly vulnerable to novel pathogens (Parker and Deem 2012). She confirmed the presence of both avian malaria and pox virus in the Galapagos (Levin et al. 2009; Deem et al. 2012). Her keynote address just got increasingly fascinating. Based on genetic analysis of tissue samples extracted from museum specimens, collected between 1891 and 1906, Parker and collaborators have documented a century of incidence of *Avipoxvirus* (the avian pox agent) in the Galapagos *-*an ongoing threat to the local avifauna *-* and determined that first introduction of the pathogen occurred in the late 1890 s (Parker et al. 2011). The Parker team has characterized, in amazing detail, the patterns of parasite abundance and distribution among iconic Galapagos birds, including: lice and flies infecting doves (*Zenaida galapagoensis*) and hawks (*Buteo galapagoensis*) (Whiteman et al. 2007; Santiago-Alarcon et al. 2008); avian malaria in penguins (*Spheniscus mendiculus*) (Levin et al. 2009); microfilariae in flightless cormorants (*Phalacrocorax harrisi*) and penguins (Siers et al. 2010); as well as lice, haemosporidian parasites, and feather mites in flycatchers (*Myiarchus magnirostris*) (Sari et al. 2013). Parker’s talk reminded us that the fragile Galapagos avifauna is facing ‘accelerated’ exposure to pathogens for which endemic species might not be evolutionarily prepared to respond with innate immunity (Deem et al. 2010; Parker and Whiteman 2012). By studying the phylogeny and phylogeography of pathogens and their hosts, plus carefully selecting the bird species which coexistence is behaviorally and ecologically intertwined (for example, the Galapagos dove or mockingbird, which colonization to the islands dates back to 2 million years, and the more recent arrival of the Galapagos hawk 200,000 years ago; Parker and Whiteman 2012) Parker’s studies are of unique basic-science significance and practical conservation value.

Carlos Valle discussed the ‘female rule’ reproductive strategy in the Galapagos flightless cormorant (*Phalacrocorax harrisi*), a newly diverged species (2 million years ago within the world’s Phalacrocoracidae), which is endemic to the islands and of singular morphology, behavior, and breeding system (Kennedy et al. 2009). Females lead courtship behavior, actively compete for mates, regulate time of their own desertion as mates, and copulate with consecutive partners (Valle 2009, 2013). Such facultative sequential polyandry and synchronization of desertion allow females to reduce brood-size to a single young, opportunity for re-mating, and increase chances for re-nesting (Valle 2009). Why do males not desert? Theoretically, male desertion could be constrained by male-based sex ratio, male-nest territoriality, asymmetry in the time males or females regain reproductive state, plus males could increase fitness by opportunistically inseminating females prior to their departure (Valle 2009). But Valle explained that unique features characterize the Galapagos cormorant reproduction: males, who are 40% larger than females, are better food providers and can feed single-young broods even when food is limited, a trait that entraps them in a ‘cruel bind’ (Trivers 1972) to caring alone for the young while the female opportunistically deserts (Valle 2013). However, female desertion, which appears also facultative (influenced by but not restricted to resources abundance), occurs when chicks reach at least 2.5 months of age; thus females have evolved mechanisms to assess optimal opportunities for desertion (that is offspring size and development, minimized threat to nests by predators when offspring approach independence) without compromising their own fitness (Valle 2009, 2013). Valle concluded that the evolution of this singular breeding system in the Galapagos flightless cormorant is likely the consequence of multiple life-history factors closely associated with drastic environmental change (that is, cyclic El Niño events; Valle 2009).

To close the behavior and environment session, Forest Rohwer discussed the captivating complexity of coral reef holobionts (that is, ecological units composed of macro-organisms and the viruses, microbes, and protists living in them) and reviewed the ‘for’ and ‘against’ arguments supporting the ‘hologenome hypothesis’, which proposes that ecological holobionts can be selected as units. Holobionts are ‘invisible’ players which shape benthic competition on coral reefs; because their viruses and micro-organisms change rapidly in response to perturbation, understanding holobiont ecology and evolution is vital for reef conservation and restoration (Vega Thurber et al. 2009; Barott and Rohwer 2012). Microbial associations of the holobiont facilitate fixation of nutrients or protect their coral or algae hosts from pathogens by secreting antibiotic compounds, which also allows them to compete for substrate (Rohwer and Vega Thurber 2009; Barott et al. 2011; Barott and Rohwer 2012). Rohwer and collaborators have postulated that by manipulating microbes and other viral communities, algal holobionts can overgrow corals via, ultimately, viral manipulation (Rohwer and Vega Thurber 2009; Vega Thurber et al. 2009). Algal and coral holobionts crawl over each other in competition for space, thus creating a physical and retro-feeding chemical microenvironment of high complexity, where algae can persist through opportunistic infection and/or by inducing hypoxia that suffocates the coral holobiont (Barott and Rohwer 2012). Because overfishing and human-induced eutrophication promote algal proliferation, coral communities can retreat and significant biodiversity be lost (Barott et al. 2012). Thus, selection favoring algae, or corals depending on the circumstances, can carry with it entire holobionts (that is, hologenome hypothesis, above). According to Rohwer, it is reasonable to view life as a ‘viral incubator’. Viruses are the ‘winners in the game of life’ since there are more viruses on Earth than cellular organisms, plus viruses encode most of the genetic diversity/information. Paradoxically, ecological and evolutionary concepts continue to be postulated ignoring the ‘virosphere’ (Rohwer and Barott 2013). Only Rohwer could have said it so metaphorically and so truthfully.

### Genome

Roderic Guigó (University of Pompeu, Spain; member of the Encyclopedia of DNA Elements project, ENCODE) and Rasmus Nielsen (University of California Berkeley, USA) talked about the technological cutting-edge strategies to study the human genome (Guigó) and specific adaptations of humans to harsh environments in the context of sampling genetic variation (Nielsen).

Roderic Guigó’s fundamental premise when discussing the genome was that because cell biochemical regulation relies ultimately on the synthesis, processing, transport, modification, and translation of RNA, cataloguing all possible ‘phenotypic’ expressions of RNA is indispensable for understanding the ‘landscape of transcription in human cells’ (Djebali et al. 2012). The Encyclopedia of DNA Elements project (ENCODE 2013), in which Guigó is involved, has used Massively Parallel Sequencing Instruments (that is, large scale, miniaturized, and robotics-based nucleotide sequencing in multiple layers of DNA, also called next generation sequencing NGS) to unravel that 75% of the genome can be transcribed and that genes are interlaced with overlapping RNA transcripts (Ecker 2012). Thus the old idea of ‘junk DNA, or non-coding DNA sequences retained ‘ as evolutionary ‘relics’ within the genome, is essentially wrong; in fact, the vast majority of the DNA seems to be transcribed into diverse functional RNAs of catalytic properties that, although do not translate into proteins (that is, non-protein coding RNAs or ncRNAs), are essential for epigenetic gene expression and regulation (Derrien et al. 2012). Guigó prompted for a redefinition of the concept of a gene and to rethink about what constitutes the minimum unit of heredity; his team of 85 collaborators and coauthors have proposed that ‘… a transcript be considered as the basic atomic unit of inheritance, therefore ‘… the term gene would then denote a higher-order concept intended to capture all those transcripts (eventually divorced from their genomic locations) that contribute to a given phenotypic trait’ (Djebali et al. 2012). In closing, Guigó explained that some of these ncRNAs, that is, the long LncRNAs, have been implicated in cancer and neurodegeneration, highlighting their importance for human health (Derrien et al. 2012). Guigó’s keynote address was illuminating, profound.

Rasmus Nielsen introduced his keynote address to the molecular footprints of human adaptations by reminding us that modern *Homo* populations have settled in environments that differ from those in which our African ancestors evolved. Cold seasonal latitudes and high altitude have shaped characteristically our current genetic diversity. Nielsen and collaborators are studying the convergent adaptations to intermediate-to-high altitude and low-oxygen atmospheric concentration (hypoxia) among Ethiopians (1,800–3,500 m), Tibetans and Andean natives (both up to 4,000 m) (Yi et al. 2010; Huerta-Sánchez et al. 2013). These three phenotypes vary distinctively across correlated traits: hemoglobin concentration, oxygen saturation in the blood, and gene involvement in Hypoxia-inducible Factors (HIF) pathway (Huerta-Sánchez et al. 2013). In respect to each other’s phenotypes, Ethiopians have elevated hemoglobin levels, Tibetans low, and Andeans high (Scheinfeldt et al. 2012; Bigham et al. 2013). Arterial oxygen saturation is high among Ethiopians, low in Tibetans, and high in Andeans (Beall 2006). Ethiopians have one gene (BHLHE41) involved in the HIF pathway which has been positively selected to cope with high altitude, Tibetans have two (EPAS1, EGLN1), and Andeans have one (EGLN1) (Huerta-Sánchez et al. 2013). Natural selection has favored the convergent arrival at three idiosyncratic responses to hypoxia and high altitudes within a commonly shared and ancestral HIF pathway, an amazing example of ‘evolution at work’ among modern humans.

### Microbes and diseases

In this mosaic-of-topics session, speakers explored lineage differentiation in unicellular eukaryotes (protists), bacterial adaptive radiation and colonization of new host-environments, the ecology and evolution of infectious diseases, and antibiotic resistance. Avelina Espinosa (affiliation above), Guillermo Paz-y-Miño-C (affiliation above), Paul Keim (Northern Arizona University, USA), and Fernando Baquero (Ramón and Cajal University Hospital, Spain) presented keynote addresses.

Espinosa and Paz-y-Miño-C examined the difficulty in discerning phylogenetic relations among unicellular eukaryotes, which evolution entails vertical inheritance of the genome combined with persistent horizontal gene transfer (HGT) across taxa (Paz-y-Miño-C and Espinosa 2010). Relying on microscopic behavioral analysis, color tagging of individual cells, and pair-mix-culturing *Entamoeba* varieties, Espinosa and Paz-y-Miño-C have brought clarity into the old problem of crypticity in *Entamoeba* lineages of diverse natural histories, that is, free-living/opportunistics (*E. moshkovski* Laredo), commensals (*E. moshkovski* snake), or parasitic (*E. invadens* IP-1, *E. invadens* VK-1: NS, *E. terrapinae*, *E. histolytica*) (Espinosa and Paz-y-Miño-C 2012). Indeed, in their lineage-discrimination laboratory experiments, clusters of trophozoites from each *Entamoeba* aggregated at a distinctive rate, density of cells per cluster, and distance among clusters. Even when grown in mixed *Entamoeba* cultures (of similar or distinctive natural histories, above), trophozoites aggregated only with members of their own lineage, which is a remarkable discrimination ability at the protist level (Espinosa and Paz-y-Miño-C 2012). Espinosa and Paz-y-Miño-C emphasized that unraveling phylogenetic relations among unicellular eukaryotes, often confounded by HGT, extinctions or highly variable genetic distances, helps us understand the environmental complexity in which vast unicellular diversity originated. Their studies linking behavior with lineage ancestry in protists are particularly important at times when prevalent large-scale molecular sampling of Earth’s life continues to unmask new organisms, which behavioral diversity - hidden in ‘crypticity’ - continues undervalued.

Paul Keim used the example of the bacteria *Yersinia pestis* (plague) to discuss ‘high fitness’ in pathogens. Highly fit, clonal populations of *Yersinia* can emerge from cryptic varieties, cause outbreaks by colonizing hosts rapidly, and fade away. Historic plague pandemics of the 6th, 14th, and 19th centuries have decimated human settlements in North Africa, Europe, Asia, and Western North America (Keim and Wagner 2009; Byrne 2012). Although contemporary outbreaks are rare due to advances in public health and modern medicine, *Yersinia* infestations can still be studied in non-human hosts, useful models for understanding fluctuations between ‘quiescent phases’ of the pathogen and ‘flare outbursts’ (Salkeld et al. 2010). Keim and collaborators have an eco-evolutionary approach to examining ‘the plague’ in Gunnison’s prairie dogs (*Cynomys gunnisoni*) (Friggens et al. 2010; Busch et al. 2011). They have proposed that prairie-dog burrows act as sites for seasonal flea proliferation and exchange among all burrow occupants, thus inducing peak transmissions of *Yersinia* (Friggens et al. 2010). Significant surge in flea abundance and prevalence has been documented during plague episodes (Friggens et al. 2010). Interestingly, Keim’s team has documented differential immune response to *Yersinia* in a single population of prairie dogs in Aubrey Valley (Arizona), apparently unaffected by the bacteria (Busch et al. 2011). In summary, since *Yersinia* can invade multiple hosts (for example, humans, prairie dogs of various species, ground squirrels, mice; Friggens et al. 2010; Salkeld et al. 2010), its cryptic varieties can remain unnoticed for prolonged periods of time and emerge virulently at critical thresholds of alternate-host and flea abundance, or when the environment changes (particularly temperature), or the host’s immune system is compromised/unresponsive, or when -still unknown - multiple ecological variables interact (Salkeld et al. 2010; Williams et al. 2013).

Fernando Baquero closed the keynote addresses with a discussion on multi-level selection in the ‘microbiosphere’ as consequence of exposure to human-made antibiotics. In nature, antibiotics act as cell-to-cell communication signals involved in regulation of gene expression, virulence and bacterial recruitment (quorum sensing), and biofilm formation (Bernier and Surette 2013; Sengupta et al. 2013). Resistance to antibiotics is a cellular response to restore the natural integrity of these communication networks via the emergence of antibiotic-resistance genes (Baquero et al. 2013). Baquero and collaborators have proposed that, during the development of the resistance, multiple interconnected units interact (that is, genes, integrons, transposons, plasmids, cells, communities and microbionts, hosts), each acting as a ‘self-interested’ entity, which benefits from the next ‘higher-hierarchical unit’ (Baquero et al. 2013). Baquero’s team has rationalized over the concept of ‘introgressive descent’ to propose that the genetic material of an evolutionary unit propagates into different hosts (hierarchical units) and is replicated within them (Bapteste et al. 2012). Thus, the evolution of antibiotic resistance occurs in the context of inter/among unit interactions (that is, levels of the microbiosphere). This view differs from the classical ‘vertical descent’ perspective where the genetic material replicates inside its own lineage. Because antibiotics influence the natural stability of the units within the hierarchy (above), they can leave up-down and down-up molecular traces of evolutionary change, which are detectable via bioinformatics analyses (Bapteste et al. 2012). Recognition of these evolutionary multi-level bonds can expand our understanding of biological complexity beyond the usual genealogical scope (for example, the origin and evolution of novelties, such as resistance to antibiotics, or the emergence of new lineages via adaptive radiation; Bapteste et al. 2012; Baquero et al. 2013).

### Posters

The Summit’s 31 posters encouraged feedback among authors and promoted networking ‘Galapagos style’, with lively dialog and beyond 250 attendees. The presentations belonged to identifiable themes: biodiversity, species formation and genetic diversity (*n* = 8), behavior and ecology (*n* = 5), conservation and management (*N* = 4), evolution of morphology (*n* = 3), infectious diseases (*n* = 3), paleontology and the fossil record (*n* = 2), cell and molecular biology (*n* = 2), systematics (*n* = 2), developmental biology (*n* = 1), and history of evolution (*n* = 1).

Several of these posters were singularly interesting (see University San Francisco of Quito 2013):

- Through mtDNA barcode analysis, D. Escobar-Camacho, R. Barriga, and S. Ron (Pontifical Catholic University of Ecuador and the National Polytechnic School, Ecuador) reported both vast species diversity of Characiform fishes in the Yasuní National Park (Amazon) and frequent crypticity among apparently similar species, thus suggesting complex ecological dynamics in lineage formation and evolution;
- G. Rivas-Torres, B. Loiselle, L. Flury, and D. Rueda (University of Florida, USA, and Galapagos National Park) warned us about pervasive alteration of native *Scalesia* forests in the Galapagos as consequence of introduced *Cedrela* trees, which act as ‘key’ modifiers in species recruitment, favoring non-native plant assemblages over native flora;
- R. González-Florian and J. Muñoz-Durán (National University of Colombia) modeled ecological scenarios of social *versus* solitary carnivores and identified that reproductive suppression, a behavioral feature of social canids, can lead to low effective population size and explain why social carnivores are particularly prone to extinction;
- S. Barrera, V. Barragán, and G. Trueba (USFQ) reported comparable antibiotic resistance in samples of *Escherichia coli* isolated from both humans and Galapagos sea lions (*Zalophus californianus*) possibly due to colonization, by human *E. coli*, of the sea lion gut, and/or horizontal gene transfer of the resistance, the findings seem restricted to areas where human settlements co-occur with sea lions, possibly linked to sewage disposal into the sea;
- M. Kemp and E. Hadly (Stanford University, USA) used Quaternary fossil record to infer spatiotemporal colonization and/or extinction events of Anole lizards in the Caribbean island of Anguilla, hence contributing to the understanding of Anguilla’s past Anole biogeography;
- S. Gutiérrez and F. Brown (University of Los Andes, Colombia, and Smithsonian Tropical Research Institute, Panama) reported the phylogenetic emergence of coloniality in the tunicate order Stolidobranchia where most basal taxa in a phylogram were solitary, whereas the terminal genera *Symplegma*, *Botryllus*, and *Botrylloides* included species with different degrees of aggregation, interdependence of individuals, viviparity, and ‘intermediate forms’, thus proposing gradual and recent evolution of coloniality in Stolidobranchia;
- N. Bizzo and P. Sano (University of Sao Paulo, Brazil) uncovered passages from historic documents authored by the Italian geologist Giambattista Brocchi, who in 1817 had already questioned the idea of species stability and proposed their change over time; Bizzo and Sano also emphasized that concepts such as species transformations and transmutations had been discussed by Gregor Mendel in papers of 1866 to 1869, reminding us that pre- and co-Darwinian naturalists did address the notion of species mutability in the early to mid-19th century.

### The Lynn Margulis Center for Evolutionary Biology

The Summit was also about launching the *Lynn Margulis Center for Evolutionary Biology* (LMCEB), affiliated with USFQ, in celebration of a genuine seeker of nature’s deep mysteries. Margulis (1938–2011) provoked fascinating controversies over the origin and evolution of cells, their nuclei and organelles, via symbiotic relationships among ancient life forms that apparently merged during the Earth’s early past (Sagan 1967; Margulis 1970, 1981). Margulis co-proposed the hypothesis of GAIA (no relation to GAIAS) which suggested that the complex associations of all organisms in the planet engender a homeostatic balance, a harmonic coexistence responsible for life’s perpetuity over eons (Lovelock and Margulis 1974). Lynn lived by this principle of fruitful association and influenced the academic careers of hundreds of naturalists; she was best friend to many, perhaps to most.

Carlos Montúfar, as Provost of USFQ, and Diego Quiroga, as Director of GAIAS, expressed their support to Antonio Lazcano (affiliation above) as president of the LMCEB. Lazcano indicated to envision the Center as a recruiter of international researchers in evolution, particularly from Latin America around a potential Latin American Society for Evolution - an initiative long awaiting realization - and as a promoter of evolution literacy among all audiences. A historic board meeting of the LMCEB took place the evening of 2 June at the brand new Galapagos Science Center facilities just built by USFQ-GAIAS in partnership with the University of North Carolina at Chapel Hill, USA. These laboratories shall attract scientific proposals from all latitudes and USFQ anticipates that the LMCEB shall sponsor research and educational events not only in the USFQ’s Galapagos campus but also at its Tiputini Field Station in the Amazon, hence offering research teams and educators two fantastic destinations for evolution-related work.

## Discussion and conclusion

Why does evolution matter? It does because it is true, scientifically conceptualized around testable hypotheses. The concept of evolution offers us the only naturalistic explanation about the origin of life, its diversification, and the phenomena resulting from the interaction between life and the environment (Paz-y-Miño-C and Espinosa 2011). We have rationalized that: ‘The phenomenon of evolution is ongoing … [it precedes in billions of years the discovery of its reality]… and it shall continue, with comparable magnitude, in time and space. The concept of evolution is about the occurrence of evolution (i.e., the aggregation of matter, the emergence of organic compounds from simpler molecules, the formation of self-replicating macro-molecules, the encasing of chemical reactions within the boundaries of lipid-layered membranes, the formation of cells and their reproduction and differentiation, and the diversification of uni- and multicellular life) and it helps us understand and represent cognitively - via mental symbolism and abstraction - the reality of evolution. Our understanding of evolution improves with new discoveries, but the reality of evolution continues to exist regardless of our awareness and level of understanding of it’ (Paz-y-Miño-C and Espinosa 2011).

The fabulous III World Evolution Summit organized by USFQ and GAIAS was a joyful celebration of evolution’s reality, a festivity that we must keep effervescent *-* if possible for eons - in the magnificent Galapagos Islands.

## Abbreviations

AdoCbl: Adenosylcobalamin
ATP: Adenosine tri phosphate
BHLHE41: Basic helix-loop-helix family member e41 (gene)
BioLogos: Creationist proposal which goal is to merge Christianity with science
CoA: Coenzyme A
DNA: Deoxyribonucleic acid
EGLN1: Egl nine homolog 1 (gene)
ENCODE: Encyclopedia of DNA Elements project
EPAS1: Endothelial PAS domain protein 1 (gene)
FAD: Flavin adenine dinucleotide
FMN: Flavin mononucleotide
GAIA: Personification of Earth in ancient Greece
GAIAS: Galapagos Institute for the Arts and Sciences
HGT: Horizontal gene transfer
HIF: Hypoxia-inducible factors pathway
LncRNA: Long non-coding ribonucleic acid
LMCEB: Lynn Margulis Center for Evolutionary Biology
mtDNA: Mitochondrial deoxyribonucleic acid
NAD: Nicotinamide adenine dinucleotide
NADP: Nicotinamide adenine dinucleotide phosphate
ncRNA: non-protein coding ribonucleic acid
RNA: Ribonucleic acid
SAM: S-adenosyl methionine
THF: Tetrahydrofolate
TPP: Thiamine pyrophosphate
tRNA: Transfer ribonucleic acid
USFQ: University San Francisco of Quito
